# Synthesis and Antimicrobial Analysis of High Surface Area Strontium-Substituted Calcium Phosphate Nanostructures for Bone Regeneration

**DOI:** 10.3390/ijms241914527

**Published:** 2023-09-25

**Authors:** Aneela Anwar, Qudsia Kanwal, Ayesha Sadiqa, Tabassam Razaq, Iqra Haider Khan, Arshad Javaid, Safia Khan, ElSayed Tag-Eldin, Mohamed Ouladsmane

**Affiliations:** 1 Department of Chemistry, University of Engineering and Technology, Lahore 54890, Pakistan; 2 Biomedical Engineering Department, Stevens Institute of Technology, Hoboken, NJ 07030, USA; 3 Department of Chemistry, The University of Lahore, Lahore 54590, Pakistan; qudsia.kanwal@chem.uol.edu.pk (Q.K.); ayesha.sadiqa@chem.uol.edu.pk (A.S.); 4 Institute of Microbiology and Molecular Genetics, University of the Punjab, Lahore 54590, Pakistan; tabassam.phd.mmg@pu.edu.pk; 5 Department of Plant Pathology, Faculty of Agricultural Sciences, University of the Punjab, Quaid-i-Azam Campus, Lahore 54590, Pakistan; iqrahaider_khan@yahool.com (I.H.K.); arshad.iags@pu.edu.pk (A.J.); 6 Faculty of Engineering and Technology, Future University in Egypt, New Cairo 11835, Egypt; safiakhan@chem.qau.edu.pk; 7 Shandong Technology Centre of Nanodevices and Integration, School of Microelectronics, Shandong University, Jinan 250101, China; 8 Department of Chemistry, College of Science, King Saud University, Riyadh 11451, Saudi Arabia

**Keywords:** antimicrobial, bioceramics, bone regeneration, calcium phosphates, *Macrophomina phaseolina*, nanopowder, strontium

## Abstract

Continuous microwave-assisted flow synthesis has been used as a simple, more efficient, and low-cost route to fabricate a range of nanosized (<100 nm) strontium-substituted calcium phosphates. In this study, fine nanopowder was synthesized via a continuous flow synthesis with microwave assistance from the solutions of calcium nitrate tetrahydrate (with strontium nitrate as Sr^2+^ ion source) and diammonium hydrogen phosphate at pH 10 with a time duration of 5 min. The morphological characterization of the obtained powder has been carried out by employing techniques such as transmission electron microscopy, X-ray diffraction, and Brunauer–Emmett–Teller surface area analysis. The chemical structural analysis to evaluate the surface properties was made by using X-ray photoelectron spectroscopy. Zeta potential analysis was performed to evaluate the colloidal stability of the particles. Antimicrobial studies were performed for all the compositions using four bacterial strains and an opportunistic human fungal pathogen *Macrophomina phaseolina*. It was found that the nanoproduct with high strontium content (15 wt% of strontium) showed pronounced antibacterial potential against *M. luteus* while it completely arrested the fungal growth after 48 h by all of its concentrations. Thus the synthesis strategy described herein facilitated the rapid production of nanosized Sr-substituted CaPs with excellent biological performance suitable for a bone replacement application.

## 1. Introduction

Calcium phosphate (CaP) is well recognized for its usage as bone-substitute materials such as fillers in bone defect repair, injectables, as reinforcement material in biocomposites and biocompatible coatings on metallic implants [1,2]. The chemical composition of biological apatite is chemically analogous to that of synthetic hydroxyapatite (HA). It is extensively employed in bone tissue replacement and augmentation with slow resorption [3,4,5]. Hydroxyapatite [Ca_10_(PO_4_)_6_(OH)_2_] is the fundamental mineral component of bones and teeth and has a tendency to join physiochemically with bone to stimulate its growth, crucial for osseointegration in bone implants. Osseointegration is required to restore bone damage and enhance implant proficiency [6]. Physiochemical and biological characteristics of HA could be enhanced by substituting Ca^2+^ ions with some other cations. Nearly all biological apatites are non-stoichiometric due to the presence of small components of cations e.g., Na^+^, Zn^2+^ Sr,^2+^ Mg^2+^, Mn^2+^, or anions e.g., CO_3_^2−^ or HPO_4_^2−^ employed in bone tissue replacement and augmentation with slow resorption [3,4,5]. Hydroxyapatite is able to accept alternate ions inside its crystal lattice. The substituted trace ions can influence crystallinity and lattice parameters, dissolution kinetics and surface properties [7,8,9]. 

Among the cations of group 2, which can substitute calcium in HA, significance of Sr has increased for its potential beneficial biological function. Its level in the inorganic component of bone is 320–400 mg. Medically, Sr has been proved to acquire valuable results on bone development particularly at the areas of higher metabolic rate [10]. Earlier in vitro and in vivo analyses have validated the osteogenic effect of Sr-substituted calcium phosphates. Strontium has drawn considerable attention because of its dual regularity properties. The administration of Sr not only decreases bone resorption but also it stimulates bone formation by stimulating osteoblast cells to secrete new bone-related matrix protein [9,11,12,13]. Furthermore, Sr has also been discovered to have anti-oesteoporosis and antiosteopenic activities in animal models, along with its antiresorptive activity [14]. It was observed that the rate of calcium absorption during bone formation could be enhanced in the presence of strontium lactate. An early clinical trial was made on patients with postmenopausal osteoporosis who had been given 1.75 g/day Sr (as strontium lactate) starting from 3 months up to 3 years. A significant increment in bone mass without any side effect was observed under the influence of strontium lactate [15].

Strontium treatment increased bone mineral content, bone volume, bone mineral density and microarchitecture. It was reported that Sr treatment minimized the risks of bone fracture. It amended the mechanical functioning of entire bone without altering the expected material characteristics of the hard tissue [16,17,18,19,20]. 

Strontium can be substituted in hydroxyapatite by replacing the calcium ion. The resultant product have higher solubility than pure HA. This is because of the larger ionic radius of the Sr^2+^ ion in comparison with calcium ion, which ultimately leads to the perturbation of the crystal lattice [21,22]. The solid products, which have previously been produced via treatment at high temperatures or hydrothermal methods, exhibit a linear deviation in the lattice parameters, with range of compositions [23,24,25]. Strontium-substituted calcium phosphates (Sr-CaP) have been reported for their antimicrobial properties [26,27]. In this study, the nanoproduct was used as antibacterial and antifungal agent against various bacterial species and fungal strain. This soil-born fungus is an opportunistic human pathogen and also a pathogen of more than 500 plant species. It causes lung and skin diseases in humans and causes root and stem rot, seedling blight, and pod and seed infections in plants [28,29].

To our knowledge, nanosized Sr-substituted calcium phosphate using single-step continuous microwave-assisted flow synthesis (CMFS) procedure has not been found in literature. All literature findings on continuous production of CaPs are very time consuming or energy demanding and comprised of high pressure and temperature conditions [30,31]. Additionally, the conventional room temperature wet precipitation procedures are very lengthy (take 12 h or more) [32,33]. In this study, novel continuous flow synthesis with microwave addition is effectively utilized to produce phase pure and ion substituted nanobioceramics with distinct compositional and physical qualities (ion substitution level, phase composition, particle size, and crystallinity), which have unique properties that could be useful in the production of various high quality nanoscale bioceramics with better antimicrobial activities for future applications. 

## 2. Results and Discussion

**Figure 1:**
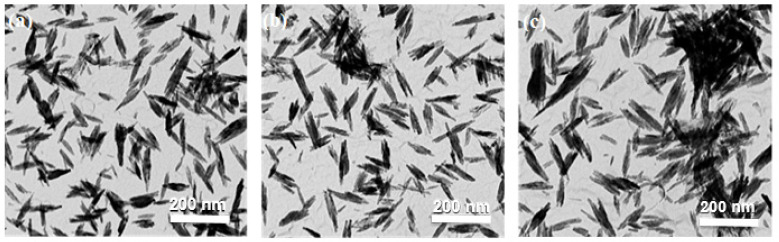
TEM images of nanoproducts made via CMFS system (**a**) 5Sr-CaP, (**b**) 10Sr-CaP, and (**c**) 15Sr-CaP.

The novel CMFS system was effectively used for the successful substitution of cations (Sr^2+^) in calcium phosphate bioceramics. TEM images had been generated to examine the morphology of the particle with changes in phase or with increasing strontium content. However, 5Sr-CaP exhibited well separated nanorods fabricated by using precursor solutions of calcium nitrate, strontium nitrate, and diammonium hydrogen phosphate, the size of these nanorods was size ~ 80 ± 15 nm beside the larger axis and ~10 ± 5 nm (200 nanoparticles sampled), beside the shorter axis as shown in Figure 1a. By further increasing Sr concentrations, longer nanorods were obtained shown in Figure 1b,c for 10Sr-CaP [~85 ± 16 nm] and 15Sr-CaP [95 ± 19 nm] beside the larger axis while ~16 ± 4 nm beside shorter axis, respectively.

The Introduction of strontium ions into the Pure CaP(HA) was demonstrated to result in an enlargement of particle size along both the a, and c-axis as shown in Table 1. Thus, the smaller (~80 ± 15 nm), well separated nanostructures were obtained by using less amount of Sr (5 wt%) as shown in Figure 1a. By increasing Sr concentration from 5 to 15 wt%, larger sized more agglomerated nanoproduct appeared as described in Figure 1b,c and supported from literature [34].

**Figure 2:**
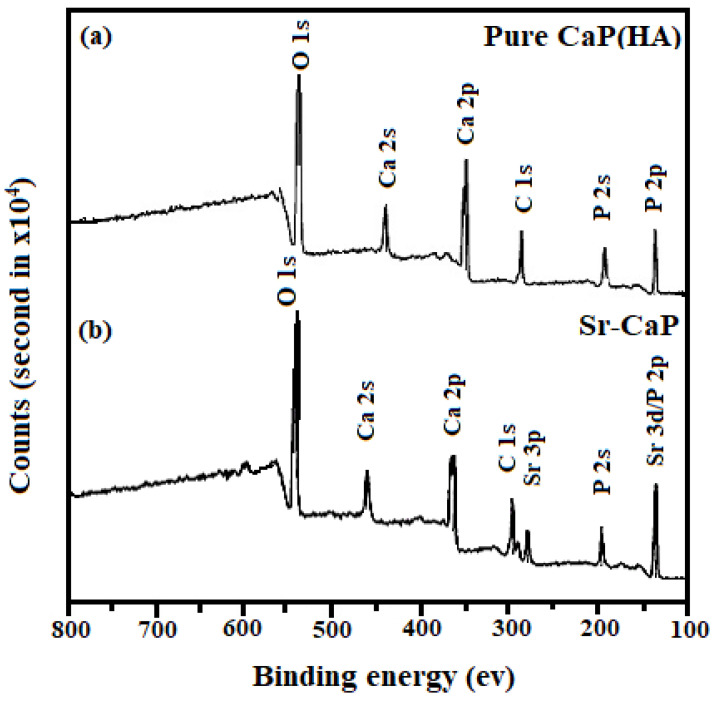
Survey spectrum of (**a**) Pure CaP(HA), and (**b**) Sr-CaP synthesized using CMFS method.

For the determination of electronic states and weight percent of Pure CaP(HA) and Sr-CaPs, XPS was performed as represented in Table 2 and Table 3 while their spectrum is shown by Figure 2. The peak at 134 eV corresponds to P 2p spectra of Pure CaP(HA). The binding energy at 532 and 347 eV are assigned to O 1s and Ca 2p, respectively [35,36]. In the examined sample, the Ca/P elemental ratio was similar to the predicted value of 1.67. The peak assigned at 134 eV is a combined peak for Sr 3d and P 2p. This is due to the smaller difference in binding energies of Sr 3d_5/2_ and Sr 3d_3/2_ (133.2 and 134.8 eV) with P 2p_3/2_ and P 2p_1/2_ peaks having binding energies of 133.4 and 134.2 eV, correspondingly. The peaks assigned at 134 eV represented the phosphate group P 2p in calcium phosphate [37]. From the data in Table 3 it was observed that the binding energy of the Sr-CaP decreases as compared to Pure CaP(HA) confirmed by the literature as well [38].

**Figure 3:**
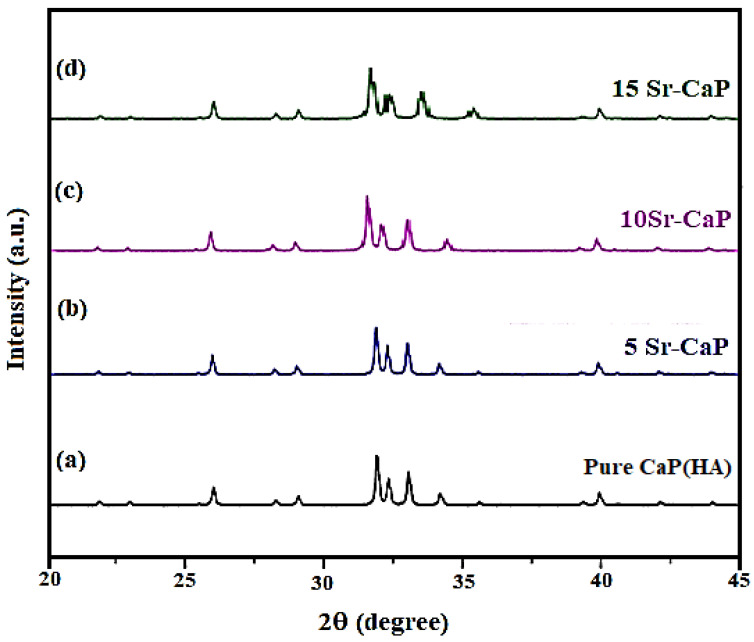
Powder XRD patterns of (**a**) Pure CaP(HA), (**b**) 5Sr-CaP, (**c**) 10Sr-CaP, and (**d**) 15Sr-CaP fabricated via CMFS system in 5 min retention time.

The powder XRD pattern of Pure CaP(HA) shown in Figure 3a revealed a distinct crystalline phase of this material. Its 2θ value experienced a slight shift from 31.8° to 31.4° as a result of Sr^2+^ ion doping. This shift also led to peak broadening, which suggests the amorphous nature of Sr-CaP. This behavior indicates that by raising the concentration of Sr, crystallinity of the HA decreases and tends to increase amorphous properties. The XRD patterns of all samples (Sr and Ca ions together) commonly revealed wider diffraction peaks with low crystallinity as revealed in Figure 3b–d.

The peak broadening is further obvious for the samples having Sr content, proposing the additional effort for Ca ion to accommodate the larger Sr ion. 

It was observed that cell parameters (a and c) slightly increased in size by increasing the strontium substitution level because of its high ionic radius compared to calcium ions [34,39]. 

The stability and surface charge of the colloidal suspension of the Pure CaP(HA) and Sr-substituted calcium phosphates were determine by measuring the Zeta potential. For orthopaedic implants to have improved surface qualities, solution stability is crucial. The colloidal suspension of the nanoproducts was evaluated with reference to their stability in aqueous solution via zeta potential. According to the previous literature, the colloids with low zeta potential tend to agglomerate more and show an incipient instability [40,41].

The higher the electrostatic repulsion, the greater the values of zeta potential and, consequently, the greater the stability of the nanoparticles in colloidal suspension. The Zeta potential for the phase of Pure CaP(HA) is −29.2 mV, whereas the corresponding values for 5Sr-CaP, 10Sr-CaP, and 15Sr-CaP are −33.5, −33.7, and −35.8 mV, respectively. A large negative surface charge has been exhibited by these nanoproducts (Table 2).

A highly stable dispersion is produced preventing the nanoproduct from agglomeration with a high zeta potential, which is demonstrated by the increased surface charge. The higher negative zeta potential may support the high adsorption rate in bone cells as well as extracellular matrix. However, in protein molecules with negative surface potential, this effect may lead to the higher charge repulsion [42,43]. 

Table 2 displays the measured Sr contents and their influence on BET surface area. By increasing the concentration of strontium the surface area of the Sr-CaP decreases, as the ionic radius of strontium is greater as compared to calcium. It was comprehended that the measured contents of Sr in the final yield were reduced as compared to the original amount added. However, the particle size of the obtained nanoparticles was increased as represented in Table 1.

**Figure 4:**
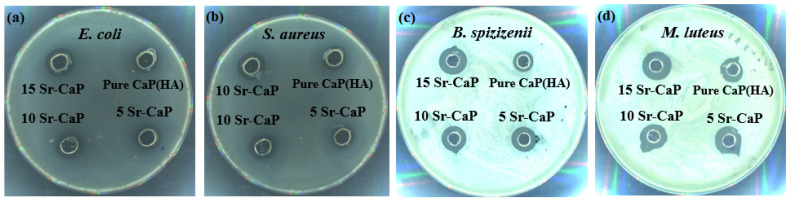
Antibacterial assay of various concentrations of Sr-CaPs against (**a**) *E.coli*, (**b**) *S. aureus*, (**c**) *B. spizizenii*, and (**d**) *M. luteus*.

The antibacterial properties of the all the samples were evaluated against four bacterial strains (*E. coli, S. aureus, B. spizizienii* and *M. Luteus*) and very promising results were obtained as displayed in Figure 4. These are the common prokaryotic bacteria causing various infections both in human and animals. 

From the results in Table 4, it can be inferred that the synthesized nanoparticles were more active against the three Gram-positive bacterial strains as compared to *E. coli* that is gram-negative. Maximum activity was shown by 15Sr-CaP against *M. Luteus* (21.7 mm), which was more than the bacterial inhibiting ability of the commonly used antibiotic vancomycin, while very close activity was exhibited in comparison with Penicillin G Sodium. 

The bioactivity of calcium phosphate hydroxyapatite improves with the introduction of strontium [44,45]. A significant difference of antibacterial activity (*p* < 0.05) was observed by statistical analysis. From Table 4, it can be clearly seen that by increasing the concentration of Sr, the antibacterial activity was increased confirming the studies of the previous literature [46]. In previous literature antibacterial activity of the strontium-substituted hydroxyapatite has been determined against *E. coli* and *S. aureus* while here in our study two more bacterial strains named *B. spizizenii* and *M. Luteus* have been used and very positive results were depicted against *M. Luteus* also showing the novelty. It has been suggested by various authors that altering the structure, chemical composition, and crystallinity of calcium phosphates could substantially improve its characteristics. The alteration in the calcium/phosphorus proportion caused by the addition of ions has a direct impact on the biocompatibility, and osteoconductivity, as it accelerates the rate of breakdown in the physiological environment [27,42]. Therefore, the cellular activity of the pathogens ceased due to electrostatic interaction between the bacterial cell wall and nanoparticles. As a result of this binding, the diffusion of nanoparticles into the bacterial cell make it easier to terminate the cell activities. 

Fungal growth appeared in all the concentrations of control after 24 h. On the contrary, there was no fungal growth in any of the concentration of fungicide mancozeb even after 96 h of incubation. All the concentrations of pure HA caused complete inhibition of the fungal growth after 24 h. A concentration of 0.5 mg/mL and above also arrested fungal growth after 48 h of incubation. The effect of 5Sr-CaP on fungal growth was similar to that of pure HA. However, 15Sr-CaP showed pronounced antifungal activity and completely arrested fungal growth for 48 h by all of its concentrations. The fungus was also unable to grow in a concentration of 0.5 mg/mL and above of this compound after 72 h (Table 5). The fungus becomes inactive as the saturation of solution increases, its ability to adhere to fungal hyphe due to high density increases. The nanoparticles inhibit the growth of fungus by disrupting cell membranes and walls, hindering the mycelial growth and conidial germination, and by developing reactive oxygen species [47,48].

Recent research has concentrated on microwave synthesis as a rapid and economical method for addressing the added benefits of full volume heating with effective energy transformation [49,50]. However, its applications are restricted to small-scale synthesis because of low penetration power [51]. Our lab has recently reported a unique and quick fabrication process for the quicker manufacturing of nanoscale pure and Sr-substituted calcium phosphates with improved nucleation rate. This study is an extension of our challenging efforts in the realm of nanotechnology [7,33,52,53]. The new CMFS process in combination with continuous flow synthesis with microwave supplement produced extremely good results. This study offered the most efficient approach to make very fine nanorods of pure and ion substituted bioactive nanobioceramics in comparatively shorter time period of just 5 min with very high surface area. The introduction of Sr in calcium alters the zeta potential, which led to the high antibacterial and antifungal activity. The results evidently confirm that strontium contents in CaPs would lead to enhanced antimicrobial activity as compared to unsubstituted calcium phosphates [42].

The ability to produce high stability smarter nanoparticles with slight changes in reactor parameters highlight that the continuous method of synthesis with microwave addition will be an esteemed addition to the synthesis literature of such nanomaterials. Furthermore, we believe this unique flow process will have applicability for the rapid manufacture of other metal-doped calcium phosphates and composite materials for future endeavour. Thus, the procedure used in this study is a single step, faster route for the synthesis with high stability, smaller size, and good biological properties.

## 3. Materials and Methods

### 3.1. Synthesis of Strontium-Substituted Calcium Phosphates

Strontium-substituted calcium phosphates were successfully made by using continuous microwave-assisted flow synthesis, as described previously [7,52]. In the synthesis procedure, the starting solutions of calcium nitrate (Sigma-Aldrich, Dorset, UK) with strontium nitrate (Sigma-Aldrich, Dorset, UK) (overall concentration of 0.5 M with Ca + Sr/P ratio of 1.67) and diammonium hydrogen phosphate (VWR International, UK) (0.3 M) were propelled to encounter in a T-piece. The pH of both the solutions was kept above 10 before the reaction proceeded. This initial mixture was joined to an 8 m Teflon coil set into an 800 W domestic microwave oven ((Samsung ME-732K, Seoul, Republic of Korea), where the response took place within 5 min. The percentage purity of all the raw materials used was 99%, and they were purchased from Sigma-Aldrich.

The nanoproduct was collected as a fine suspension at the end, washed with deionized water followed by centrifugation for 5 min. The washed solid product was dehydrated at a temperature of 75–80 °C in oven for 12 h. The final product was achieved as a fine white nanopowder with a percentage yield of about 85. The samples were marked as Pure CaP(HA), 5Sr-CaP, 10Sr-CaP, and 15Sr-CaP corresponding to 0, 5, 10, and 15 expected strontium weight ratio in the samples.

### 3.2. Characterization Techniques

#### 3.2.1. Transmission Electron Microscopy (TEM)

The particle size and morphology of the synthesized nanoparticles was determined by taking images using a JEOL 1200EX Transmission Electron Microscope. In methanol 10 mg sample was dissolved by ultrasonication for two minutes to form a highly diluted solution. The finished suspension was then dispersed in a few droplets onto a copper grid that had been coated with carbon. 

#### 3.2.2. Chemical Analysis

For the determination of electronic states and weight percent of the substituted group of Sr-CaPs, a Thermo Scientific X-ray photoelectron spectrometer was used. The greater surface sensitivity of about 40–100 Å marks this technology best for the determination of elemental ratio. The spectrum for survey scans was generally taken at an energy of 150 eV. The XPS spectra were administered using CasaXPS 2.3 software.

#### 3.2.3. Powder X-Ray Difraction

A Bruker D4 Endeavour^TM^ diffractometer was utilized for XRD examination of the nanoproduct. The data for continuous scans of all the powdered samples were obtained over the 2θ from 5–70° range with the step size of 0.04° at 2 s per step by means of Cu-Kα radiation where λ = 1.5406 A°. 

#### 3.2.4. Zeta Potential Measurement

A Malvern Instruments Zetasizer was used to determine the Zeta potential of fabricated Sr-CaPs. Electrophoretic technique was used for the particle evaluation using a disposable zeta cell. Electrophoretic mobility measurement in a specific electric field demonstrated the colloidal stability of the scattered nanoparticles.

#### 3.2.5. BET Surface Area Analysis

A Brunauer–Emmett–Teller (BET) surface area analysis was performed using a multipoint BET surface area analyzer, capable of analyzing 6 samples simultaneously. The as prepared nanoproduct was precisely weighed and degassed for twelve hours at 180 °C before measurements. 

### 3.3. Antibacterial Activity

An agar well diffusion methodology has been employed for the evaluation of the antimicrobial efficacy of Sr-substituted calcium phosphates [54,55]. For antibacterial screening, *Escherichia coli* (ATCC 14169), *Staphylococcus aureus* (ATCC 29737), *Bacillus subtilis* subsp. *spizizenii* (ATCC 6633), and *Micrococcus luteus* (ATCC 4698) were used. Penicillin G Sodium (45 IU/mL) and Vancomycin (10 µg/mL) were used as reference antibiotics standards. Antibiotic Medium N° 11 (AMN-11) (30.5 g) was suspended in 1 L of de-ionized water, mixed well by heating followed by frequent agitation and complete dissolution was achieved by boiling for one minute. Sterilization of the AMN-11 was carried at 121 °C and 15 psi for 15 min in an autoclave. The concentration of all the samples was taken to be 100 µg/mL, Meteller Tolledo XPR Microanalytical balance was used to weigh the samples. In Petri plates, the sterilized AMN-11 was poured, settled, and left for 24 h. The freshly prepared inoculum suspension of each test bacteria was evenly dispersed over the surface of the petri plates containing AMN-11, then with the help of a sterilized corkborer a hole of diameter of 6 mm was punched and a volume of 100 µL reference antibiotics, and all samples were dispensed into the wells. The samples and reference antibiotics were incubated for 24 h at a temperature of 37 °C and clearance zones were measured by calibrated digital Vernier caliper. For statistical analysis of the antibacterial effect, a hypothetical pair t-test with OriginPro 9 software was performed.

### 3.4. Antifungal Bioassays

Antifungal bioassays were carried out following the procedure of Jabeen et al. [56] with little modifications. Six milligrams of each compound and reference fungicide mencozeb (active ingredients) were dissolved in 30 µL dimethyl sulphoxide (DMSO). Each of the dissolved compound and fungicide was mixed in autoclave (cooled at room temperature) malt extract broth to make a total volume 3 mL. This amount was split into two 1.5 mL-sized parts. One portion was divided into three sub-portions of 0.5 mL each for antifungal bioassays. To the other portion, 1.5 mL autoclaved malt extract broth was added and repeated the previous step. Likewise, a series of dilutions were prepared to have a concentrations range of 2 to 0.0156 mg/mL. Similarly, a set of control treatments was prepared to maintain the same amount of DMSO for comparison. Bioassays were carried out by adding 10 µL suspension of *M. phaseolina* in each test tube containing 0.5 mL growth medium. The tubes were monitored for 96 h with intervals of 24 h to observe the appearance of fungal growth. 

## 4. Conclusions

Pure and ion substituted calcium phosphates were successfully prepared using a single step, continuous flow system from calcium phosphate ion solutions in just a five minute retention time. Vigilant control of the reaction parameters used resulted in high surface area, nanoscale calcium phosphate rods compared to traditional synthesis process. The synthesized nanoparticles are highly stable with a zeta potential value of −35.8 mV and the 15Sr-CaP nanoparticles have exhibited a very strong antibacterial activity against *M. luteus* and antifungal activity against *M. phaseolina* confirming their biocompatibility and bioactivity. Thus, the acquired nanoproduct with its distinctive features is a providential material with pronounced antimicrobial properties and has numerous potentials to be used in bone replacement applications.

## Figures and Tables

**Table 1:** Structural morphology of Pure CaP(HA), and various concentrations of Sr-CaPs.

Sample Identity	XRD Crystallite Morphology		TEM Particle Size	XPS Ca+Sr/P
	a (Å)	c (Å)	nm	
Pure CaP(HA)	9.41 ± 0.002	6.81 ± 0.012	75	1.67
5Sr-CaP	9.42 ± 0.003	6.89 ± 0.012	80	1.65
10Sr-CaP	9.43 ± 0.004	6.92 ± 0.012	85	1.63
15Sr-CaP	9.45 ± 0.004	6.95 ± 0.012	95	1.61

**Table 2:** Sample identification using XPS analysis, zeta potential, and BET surface area measurements.

Sample ID	Sr Added (wt%)	Sr Measured (wt%)	Zeta Potential (mV)	BET Surface Area (m^2^ g^−1^)
Pure CaP(HA)	0	0	−29.2 ± 0.2	198 ± 2.30
5Sr-CaP	5	3.4 ± 0.11	-33.5 ± 0.3	165 ± 1.92
10Sr-CaP	10	7.5 ± 0.17	-33.7 ± 0.1	157 ± 1.63
15Sr-CaP	15	11.7 ±0.09	-35.8 ± 0.2	136 ± 1.03

**Table 3:** The binding energy of Pure CaP(HA) and Sr-CaP.

	**Binding Energy (eV)**					
	**O 1s**	**Sr 3d_3/2_**	**Ca 2s**	**P 2p_3/2_**	**P 2s**	**P 2p**
Pure CaP(HA)	532	-	440	347	191	134
Sr-CaP	530	134	439	345	189	134

**Table 4:** Antibacterial evaluation of Pure CaP(HA) and various concentrations of Sr-CaPs against reference antibiotic standards (Vancomycin and Penicillin G Sodium) and four bacterial strains.

Bactria	Zone of Inhibition (mm)					
	Vancomycin	Penicillin G	Pure CaP(HA)	5Sr-CaP	10Sr-CaP	15Sr-CaP
*E. coli*	18.09 ± 0.04	23.67 ± 0.03	3.34 ± 0.06	5.89 ± 0.11	6.23 ± 0.04	7.42 ± 0.23
*S. aureus*	16.52 ± 0.03	21.03 ± 0.11	5.99 ± 0.04	12.65 ± 0.07	12.74 ± 0.04	13.98 ± 0.21
*B. spizizenii*	15.27 ± 0.08	19.99 ± 0.06	5.89 ± 0.08	8.24 ± 0.09	8.63 ± 0.04	11.94 ± 0.12
*M. luteus*	19.54 ± 0.06	22.91 ± 0.14	6.67 ± 0.02	13.96 ± 0.03	18.33 ± 0.04	21.71 ± 0.09

**Table 5:** Minimum inhibitory concentrations (MIC) of Pure CaP(HA), 5Sr-CaP, 15Sr-CaP, and reference fungicide mancozeb against opportunistic human pathogen *Macrophomina phaseolina*.

NPs	Time	Minimum Inhibitory Concentration MIC (mg/mL)							
	(h)	2	1	0.5	0.25	0.125	0.0625	0.0312	0.0156
Control	24	+	+	+	+	+	+	+	+
	48	+	+	+	+	+	+	+	+
	72	+	+	+	+	+	+	+	+
	96	+	+	+	+	+	+	+	+
Mancozeb	24	−	−	−	−	−	−	−	−
	48	−	−	−	−	−	−	−	−
	72	−	−	−	−	−	−	−	−
	96	−	−	−	−	−	−	−	−
Pure CaP(HA)	24	−	−	−	−	−	−	−	−
	48	−	−	−	+	+	+	+	+
	72	+	+	+	+	+	+	+	+
	96	+	+	+	+	+	+	+	+
5Sr-CaP	24	−	−	−	−	−	−	−	−
	48	−	−	−	−	+	+	+	+
	72	+	+	+	+	+	+	+	+
	96	+	+	+	+	+	+	+	+
15Sr-CaP	24	−	−	−	−	−	−	−	−
	48	−	−	−	−	−	−	−	−
	72	−	−	−	+	+	+	+	+
	96	+	+	+	+	+	+	+	+

− No fungal growth; + Fungal growth appeared.

## Data Availability

Not applicable.
